# Correction to “Pediatric Floor of Mouth Lipoma: A Rare Case Report”

**DOI:** 10.1002/ccr3.71441

**Published:** 2025-11-11

J. Awan, S. Masood, A. Javaid, F. Shahid, and R. G. “Omarzai Rehman A Pediatric floor‐of‐mouth lipoma: a rare case report,” Clinical Case Reports 13, no. 10 (2025): e71238. https://doi.org/10.1002/ccr3.71238.

We failed to add a new author that was introduced at the revision stage and who contributed extensively to the revised manuscript of the paper. The new author is Sadia Chaudhry, and the new author order is as below:

1. Javeria Awan
2. Sundas Masood
3. Sadia Chaudhry
4. Amna Javaid
5. Fatima Shahid
6. Rahmat Gul Omarzai
7. Abdur Rehman

We apologize for this error.

